# SGLT2 inhibitor improves the prognosis of patients with coronary heart disease and prevents in-stent restenosis

**DOI:** 10.3389/fcvm.2023.1280547

**Published:** 2024-01-11

**Authors:** Qing Zhang, Zhiwen Deng, Tudi Li, Kaitong Chen, Zhihuan Zeng

**Affiliations:** Department of Cardiovascular Diseases, The First Affiliated Hospital of Guangdong Pharmaceutical University, Guangzhou, Guangdong Province, China

**Keywords:** SGLT2 inhibitor, restenosis, coronary heart disease, AMI—acute myocardial infarction, ASCVD-atherosclerotic cardiovascular disease

## Abstract

Coronary heart disease is a narrowing or obstruction of the vascular cavity caused by atherosclerosis of the coronary arteries, which leads to myocardial ischemia and hypoxia. At present, percutaneous coronary intervention (PCI) is an effective treatment for coronary atherosclerotic heart disease. Restenosis is the main limiting factor of the long-term success of PCI, and it is also a difficult problem in the field of intervention. Sodium-glucose cotransporter 2 (SGLT2) inhibitor is a new oral glucose-lowering agent used in the treatment of diabetes in recent years. Recent studies have shown that SGLT2 inhibitors can effectively improve the prognosis of patients after PCI and reduce the occurrence of restenosis. This review provides an overview of the clinical studies and mechanisms of SGLT2 inhibitors in the prevention of restenosis, providing a new option for improving the clinical prognosis of patients after PCI.

## 1 Introduction

Coronary heart disease (CHD) is a narrowing or obstruction of the vascular cavity caused by atherosclerosis of the coronary arteries, which leads to myocardial ischemia and hypoxia. With the change in human lifestyle, the number of people with disorders of glucose and lipid metabolism has increased greatly, as well as the incidence of CHD has increased (1). With the progressive implementation of guidance-supported treatments for coronary heart disease, such as percutaneous coronary intervention (PCI), antiplatelet therapy, statins, and angiotensin converting enzyme (ACE) inhibitors, the overall prognosis for CHD has improved significantly over time. Percutaneous coronary intervention therapy for CHD has the advantages of less trauma, fast postoperative recovery, and fewer postoperative complications (2, 3). It can unclog the infarcted vessel, achieve coronary artery reperfusion, and recover the infarcted myocardium to the greatest extent, thus significantly improving the clinical symptoms of patients and increasing the long-term survival rate of patients with coronary heart disease (4). Although medications and interventional treatments greatly reduce the risk for mortality in patients with coronary heart disease, they are still at high risk of developing chronic heart failure (HF) and subsequently facing a higher risk of death and disability (5–8). After the first anniversary of PCI, dual antiplatelet therapy, cardiac rehabilitation, and cardiologist follow-up have typically ended, and the use of secondary prevention medications may be declining (8). It may lead to restenosis, recurrent myocardial infarction (MI), and other manifestations of cardiovascular disease, such as stroke. However, improvements in prognosis for coronary heart disease, especially for myocardial infarction, have slowed in recent years due to limited new treatment options. Therefore, new therapeutic approaches are needed to further improve the treatment of patients with myocardial infarction and prevent recurrence of cardiovascular events.

**Figure 1:**
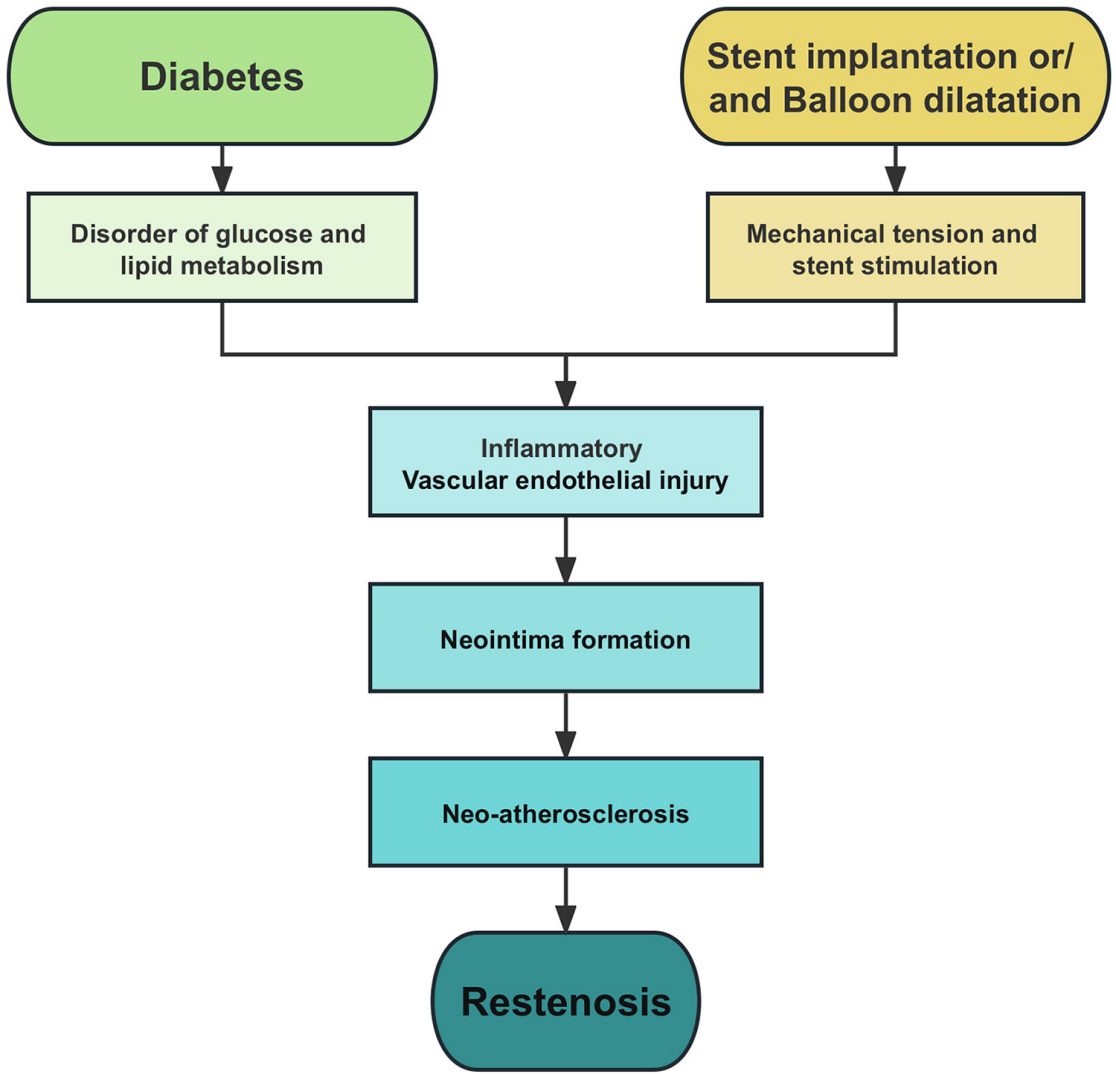
Pathophysiology of restenosis. Endothelial injury, inflammation, and neointima formation are the main causes of restenosis.

Restenosis is one of the main causes of recurrent cardiovascular events after PCI, and it is also a difficult problem in the field of intervention (9). Although the availability of drug-eluting stents (DES) has significantly reduced the rate of restenosis and revascularization, the incidence of intrastent restenosis (ISR) is still as high as 5%–10% (10). Therefore, prevention of restenosis has important clinical significance in improving the prognosis of CHD patients treated with PCI. Clinically, insulin resistance and endothelial dysfunction in diabetes mellitus (DM) patients make them more prone to be complicated with coronary heart disease. Patients with DM have an increased incidence of stent thrombosis and restenosis after PCI compared with non-DM patients (11–13). The main mechanisms of restenosis are summarized in Figure 1.

SGLT2 inhibitor is a new type of oral glucose-lowering agent used in the treatment of diabetes in recent years (14). They mainly combine with SGLT2 in the renal tubules to prevent the reabsorption of glucose by the kidney and increase the excretion of glucose in the urine, thus reducing the plasma glucose level (15). In addition to glucose-lowering effects, SGLT2 inhibitor has also been shown to have significant cardiovascular protective effects (16–19). The main manifestations are reducing blood pressure, regulating cardiac energy metabolism (16), improving vascular function (15, 20), inhibiting the sympathetic nervous system (21, 22), and preventing adverse myocardial remodeling (23). In addition, there is growing evidence that the use of selected SGLT2 inhibitors in patients with atherosclerotic cardiovascular disease (ASCVD) can improve cardiovascular outcomes (24). The 2023 ESC guidelines for the management of cardiovascular disease in patients with diabetes recommend that, along with GLP-1RAs, SGLT2 inhibitors are the preferred anti-hyperglycemic therapy for T2DM patients with ASCVD, independent of glycemic control considerations and independent of background metformin use (25). SGLT2 inhibitors can improve the prognosis of CHD patients undergoing PCI and reduce the occurrence of various complications, such as acute kidney injury (26, 27), lethal ventricular arrhythmias (21), and ISR (28). Previous studies have shown that SGLT2 is mainly expressed in human kidney and small intestine cells (29), but could not be detected in human myocardium (30). Recently, SGLT2 was found to be expressed in human endothelial cells (EC) and smooth muscle cells (SMC) using immunocytochemistry and western blotting techniques (31, 32). This finding may indicate that SGLT2 may be related to the occurrence and development of CHD and restenosis. This article will review the mechanisms of SGLT2 inhibitors in preventing CHD and restenosis after PCI, to provide new options for improving the clinical prognosis of CHD patients.

## 2 SGLT2 inhibitor improves the prognosis of patients with CHD

The positive effects of SGLT2 inhibitors in patients with heart failure, particularly in patients with reduced ejection fraction (HFrEF), have been demonstrated (33). ESC guidelines recommend SGLT2 inhibitors as the cornerstone of drug therapy for heart failure, regardless of whether they have combined diabetes (34). Recent clinical trials, including CVD-REAL Nordic (35), EMPA-REG OUTCOME (36–38), and the CANVAS project (39, 40), confirmed the effectiveness of SGLT2 inhibitors in patients with coronary heart disease.

A meta-analysis of six SGLT2 inhibitor trials demonstrated a reduction in the primary ASCVD based composite of time to first MACE (24). This was most apparent in patients with established ASCVD. Compared with the use of other glucose-lowering drugs, the use of SGLT2 inhibitors is associated with reduced major adverse cardiovascular events (MACE) and exert cardiovascular protection in patients who underwent PCI (Table 1) (41, 42).

**Table 1:** Clinical trial of SGLT2 inhibitor, post-PCI.

Author	Study design	Patients, *n*; group	Primary endpoint	Follow-up	Result
Marfella et al. (28)	An observational, prospective study	Total: 377 patients with T2DM and AMI undergoing PCI. Never SGLT2i users (*n* = 200). Current SGLT2i users (*n* = 177)	MACE defined as cardiac death, re-infarction, and heart failure related to ISR	1 year	The incidence of ISR-related MACE was higher in never SGLT2i-users (22.1%; *n* = 44) compared with SGLT2i-treated patients (10.2%; *n* = 18) (HR = 0.418, 95% CI = 0.241–0.725, *P* = 0.002)
Zhu et al. (42)	A single-center retrospective analysis study	Total: 786 patients with AMI undergoing PCI. DAPA-free (*n* = 645). DAPA (*n* = 141)	MACE including overall deaths, heart failure, nonfatal MI, nonfatal stroke, and unplanned repeat revascularization (URR)	Median follow-up of 23 months	The incidence of MACE: 118 (18.3%) in the DAPA-free group and 12 (8.5%) in the DAPA group). DAPA was significantly associated with the reduced risk of MACE (HR = 0.170, 95% CI = 0.078–0.373, *p* < 0.001)
Hashikata et al. (98)	An open labeled, single-center, randomized (1:1), two-arm clinical trial	Total: 28 diabetes patients with CAD planned for DES stenting. empagliflozin group (*n* = 15); oGLD group; *n* = 13)	Thickeness of neointimal tissue at 12-month follow-up after stenting, which was evaluated as the mean NIH thickness (NIT) using OCT	12 months	In OCT analysis, neointima was significantly less in the empagliflozin group than the oGLD group (mean NIH thickness: 137 ± 32 vs. 168 ± 39 μm, *p* = 0.02)
Liu et al. (23)	A retrospective cohort study	Total: 188 patients with anterior wall STEMI who received emergency PCI Control group (*N* = 96) Dapagliflozin group (*N* = 82)	The in-crease in LVEDV of echocardiography ≥20% after 6 months compared to the period of admission was regarded as the standard of ventricular remodeling	6 months	The reduction of LVEDV 6 months after admission in the dapagliflozin group was significantly higher compared to the control group (*P* < 0.01); the increase in LVEF 6 months after admission in the dapagliflozin group was higher than that in the control group (*P* < 0.01)
Paolisso (43)	Multicenter international registry	Total: 646 diabetic AMI patients undergoing PCI. SGLT2-I users (*n* = 111). Non-SGLT-I users (*n* = 535)	A composite of cardiovascular death, recurrent AMI, and hospitalization for HF	Median follow-up of 24 ± 13 months	After adjusting for confounding factors, the use of SGLT2i was identified as independent predictor of reduced MACE occurrence (HR = 0.57; 95% CI: 0.33–0.99; *p* = 0.039) and HF hospitalization (HR = 0.46; 95% CI: 0.21–0.98; *p* = 0.041)

ISR, intrastent restenosis; MACE, major adverse cardiovascular events; DAPA, dapagliflozin; OCT, optical coherence tomography; oGLD, other hypoglycemic drugs; LVEDV, left ventricular end-diastolic volume; LVEF, left ventricular ejection fraction; AMI, acute myocardial infarction; HF, heart failure.

### 2.1 Improve the prognosis of patients with myocardial infarction

SGLT2-I AMI PROTECT (NCT05261867) (43) enrolled 646 AMI patients with DM undergoing PCI. The patients were classified into SGLT2 inhibitors users and non-SGLT2 inhibitors users according to the glucose-lowering treatment at admission. The research results show that the use of SGLT2 inhibitors was associated with a significantly reduction in-hospital cardiovascular death, arrhythmic burden, and incidence of contrast media-induced acute kidney injury. After adjusting for confounders, the use of SGLT2 inhibitors was identified as an independent predictor of reduced incidence of MACE and HF hospitalization (44).

EMMY trial (NCT03087773) (45) is a randomized, controlled, double-blind trial to evaluate the role of empagliflozin on cardiac function in patients with acute myocardial infarction (AMI). In this multicenter study, there are 237 patients with AMI received 10 mg once daily of empagliflozin within three days after PCI, while 239 patients received a placebo. The primary outcome of the reduction in N-terminal pro-B-type natriuretic peptide (NT-proBNP) was significantly higher in the empagliflozin group alongside the absolute left ventricle ejection fraction also great improving with those receiving empagliflozin. This suggests that SGLT2 inhibitor has the potential to improve recovery after PCI and encourages related studies in patients with CHD.

EMPACT-MI trial (NCT04509674) (46) was a randomized, double-blind, placebo-controlled, multicenter trial designed to evaluate the efficacy of empagliflozin 10 mg daily in patients at high risk of new heart failure after acute myocardial infarction. Because previous clinical trials of SGLT2 inhibitors excluded patients with acute or recent myocardial infarction, the efficacy of these drugs in reducing future cardiovascular risk in the post-myocardial infarction population was unclear. The EMPACT-MI trial will further evaluate the safety and efficacy of empagliflozin in people at high risk of heart failure or death after myocardial infarction.

DAPA-MI trial (NCT04564742) (47) is another ongoing parallel, randomized, double-blind, placebo-controlled, multicenter phase 3 trial in patients with AMI. Patients with chronic symptomatic heart failure with a known reduced left ventricular ejection fraction (LVEF ≤40%) were excluded from the trial because these patients had an absolute indication for treatment with an SGLT2 inhibitor. This trial evaluated the effect of 10 mg dapagliflozin administered once daily compared with placebo in preventing death, heart failure or other adverse cardiovascular and metabolic events outcomes. The trial explores opportunities to further improve outcomes in patients with impaired left ventricular function after myocardial infarction, which may broaden the indication for the use of SGLT2 inhibitor trials in patients with acute myocardial infarction are listed in Table 2.

**Table 2:** Description of SGLT2 inhibitor trials in patients with acute myocardial infarction.

Trial	Intervention	Study population	Primary outcome	Secondary outcomes	Aims or conclusion
SGLT2-I AMI PROTECT (NCT05261867)	Observational registry chronic SGLT2-I therapy vs. non-SGLT2-I users	Patients (*n* = 583) with DM and AMI undergoing PCI	The following inflammatory markers were evaluated at different time points: white-blood-cell count, NLR, PLR, NPR, and CRP. Infarct size was assessed by echocardiography and by peak troponin levels		Type 2 diabetic AMI patients receiving SGLT2-I exhibited significantly reduced inflammatory response and smaller infarct size, independently of glucose-metabolic control
EMMY (NCT03087773)	Empagliflozin 10 mg vs. matching placebo once daily	Patients (*n* = 476) with AMI accompanied by CK >800 IU/L, with or without DM	Change in NT-proBNP levels from randomization to week 26	Changes in NT-proBNP levels from randomization to Week 6, echocardiographic parameters, ketone body, glycated hemoglobin, body weight	Among AMI patients, early initiation of empagliflozin resulted in a significantly greater median NT-proBNP reduction than with placebo over 26 weeks
EMPACT-MI (NCT04509674)	Empagliflozin 10 mg vs. placebo once daily	Patients with MI must have new signs or symptoms of pulmonary congestion requiring treatment or new LVEF <45%, and at least 1 additional risk factor for development of future HF	Time to first hospitalization for HF or all-cause mortality	Total HHF or all-cause mortality, Total non-elective CV hospitalizations or all-cause mortality, Total non-elective all-cause hospitalizations or all-cause mortality, Total hospitalizations for MI or all-cause mortality, Time to CV mortality	EMPACT-MI will inform clinical practice regarding the role of empagliflozin in patients after an MI with high- risk for the development of future HF and mortality
DAPA-MI (NCT04564742)	Dapagliflozin 10 mg vs. placebo once daily	Patients without known diabetes or established HF, presenting with MI and impaired left ventricular systolic function or Q-wave MI	Hierarchical composite outcome: (1) Death (first CV death, followed by non-CV death), (2) Hospitalization due to heart failure, (3) Nonfatal MI, (4) AF/flutter event, (5) New onset of T2DM, (6) NYHA functional classification at last visit, (7). Body weight decrease of at least 5% at last visit	Consist of the same composite as the primary outcome, excluding body weight reduction	DAPA-MI trial will explore opportunities to improve further the outcome of patients with impaired LV function after MI

DM, diabetes mellitus; AMI, acute myocardial infarction; CK, creatine kinase; NLR, neutrophil-to-lymphocyte ration; PLR, platelet-to-lymphocyte ratio; NPR, neutrophil-to-platelet ratio; CRP, c-reactive protein; LV, left ventricular; CV, cardiovascular; HF, heart failure.

### 2.2 Reduces the risk of repeat revascularization

A subanalysis of the EMPA-REG OUTCOME randomized trial (48) elucidates the benefit of empagliflozin in patients with type 2 diabetes and a history of CABG, suggesting that empagliflozin significantly reduces important adverse cardiovascular and renal outcomes in this high-risk population. Among participants with a history of coronary artery bypass, compared with placebo, cardiovascular death was reduced by 48%, all-cause mortality by 43%, hospitalization for HF by 50% with empagliflozin. Among participants with a history of coronary artery bypass at baseline, the proportion receiving empagliflozin (7.7%) was lower than that receiving placebo (9.8%) and had undergone ≥1 coronary artery reconstruction during the trial period. Similarly, a nationwide retrospective cohort study showed that SGLT2 inhibitors, compared with dipeptidyl peptidase-4 inhibitors, were associated with a lower risk of coronary revascularization, combined renal outcomes, and all-cause mortality in T2DM patients after PCI (41).

A prospective, observational study (28) included a total of 377 patients with type 2 diabetes who underwent PCI. Comparing those who had never used SGLT2 inhibitors, the patients who were currently using SGLT2 inhibitors with a significantly lower incidence of MACE defined as cardiac death, re-infarction, and heart failure. Cox regression analysis showed that the use of SGLT2 inhibitors also significantly reduced the incidence of ISR-related adverse cardiovascular events in patients with well-controlled blood glucose (mean HbA1c <7% within 1 year). This also suggests that the mechanism by which SGLT2 inhibitors reduces the occurrence of ISR may be independent of the glucose-lowering effect. Recently, another retrospective real-world study (49) also showed that dapagliflozin significantly improved BMI, blood pressure, and glucose outcomes in patients with T2DM combined with coronary heart disease after PCI and had a beneficial effect on MACE and ISR.

## 3 Mechanism of action of SGLT2 inhibitor in preventing coronary heart disease and restenosis

**Figure 2:**
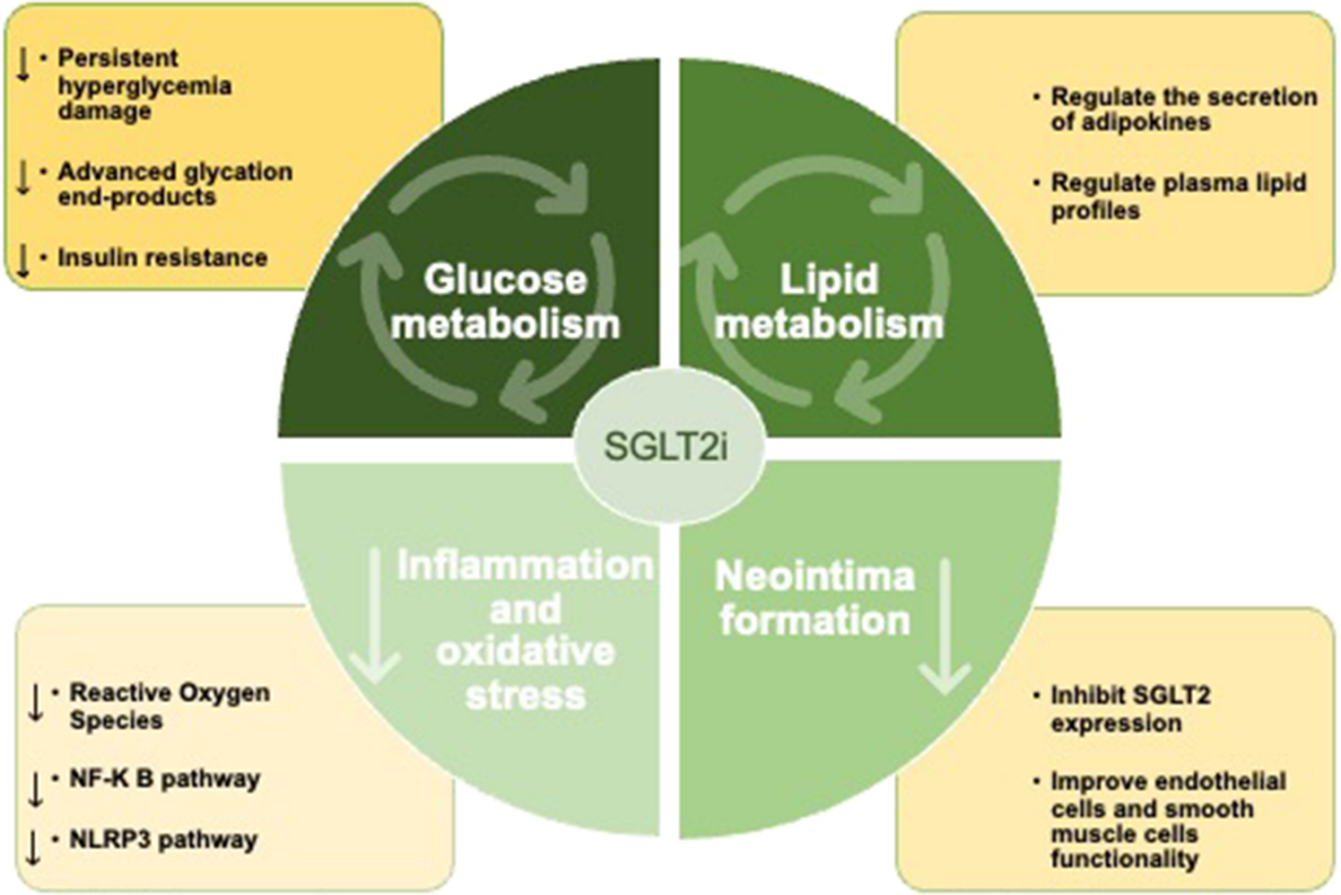
SGLT2 inhibitors can improve the prognosis of patients after PCI by improving glucose and lipid metabolism, inhibiting inflammatory response and oxidative stress, and inhibit neointima formation.

Mechanistically, inhibition of SGLT2 leads to increased renal glucose excretion and decreased serum glucose levels. With the increase in clinical application, many studies have shown that SGLT2 inhibitors can significantly improve the prognosis of CHD patients. However, it is difficult to explain the glucose-lowering effect of SGLT2 inhibitors to inhibit arteriosclerosis and long-term pathological improvement. This suggests that SGLT2 inhibitors may play a direct role. In addition to its glucose-lowering effects, several studies, including the ongoing EMPACT-MI trial (46) and DAPA-MI trial (47), have demonstrated a positive cardiovascular protective effect of SGLT2 inhibitors in non-DM patients. Interestingly, SGLT2 inhibitors could reduce ISR-related events in type 2 DM (T2DM) patients with acute coronary syndrome and the effect was independent of glycemic control, suggesting that SGLT2 inhibitors may have a beneficial effect on coronary artery remodeling after stent implantation, perhaps by modulating a wide range of metabolic, molecular, and hemodynamic mechanisms besides its glucose-lowering effects. We summarize the main mechanism in Figure 2.

### 3.1 SGLT2 inhibitor can improve glucose metabolism

Several studies have proved that diabetes is an independent risk factor for ISR in patients after PCI (11–13). DM patients have an increased incidence of restenosis after PCI compared with non-DM patients (50). The mechanism of ISR development in DM patients may be different from non-DM patients. Diabetes results in more extensive neointimal hyperplasia, atherosclerotic plaque formation, hemodynamic changes, and inadequate compensatory vascular remodeling (51–55). Faries et al. found that VSMCs of DM patients exhibit remarkably increased rates of proliferation, adhesion, and migration, as well as abnormal cell culture morphology (56). In addition, Moreno et al. found higher levels of lipid-rich atherosclerosis, macrophage infiltration, and subsequent thrombosis in coronary artery tissue of DM patients compared to non-DM patients. These differences indicate an increased susceptibility to coronary thrombosis in patients with DM (57). These observations are consistent with the clinically observed increased incidence of atherosclerosis and restenosis in patients with diabetes.

SGLT2 inhibitor is a new type of oral glucose-lowering agent used in the treatment of diabetes in recent years (14). They mainly combine with SGLT2 in the renal tubules to prevent the reabsorption of glucose by the kidney and increase the excretion of glucose in the urine, thus reducing the plasma glucose level (15). The unique glucose-lowering mechanism of SGLT2 inhibitors independent of insulin secretion makes them ideal for the treatment of T2DM.

#### 3.1.1 Reduce persistent hyperglycemia damage

The effect of persistent hyperglycemia on the development of macrovascular and microvascular complications has been widely discussed (58, 59). The harmful effects of glucose are already present when plasma glucose levels fall below the threshold for diagnosing DM (60). Hyperglycemia initially leads to an imbalance between nitric oxide (NO) bioavailability and reactive oxygen species (ROS) accumulation, which in turn leads to increased inflammatory response and oxidative stress, endothelial dysfunction, and vascular smooth muscle proliferation, ultimately leading to coronary atherosclerotic plaque formation and restenosis (59, 61).

As a new oral glucose-lowering agent, SGLT2 inhibitor can significantly reduce the levels of fasting plasma glucose and HbA1c, and alleviate the endothelial damage caused by persistent hyperglycemia (62). Studies in cultured endothelial cells have shown that high glucose increases inflammatory mediators and SGLT2 expression, and intervention with SGLT2 inhibitors can inhibit the development of unstable plaque phenotypes (63). A randomized clinical trial found that the use of SGLT2 inhibitors significantly reduced the absolute risk of atherosclerotic thrombosis in patients with T2DM (55). Joubert et al. found that the total levels of O-GlcNAcylated proteins were significantly lower in dapagliflozin-treated SKO mice than in untreated SKO mice (64). This suggests the notion that dapagliflozin partially corrects cardiac dysfunction through glucose-lowering effects. Long-term application of SGLT2 inhibitors has a good effect on the clinical prognosis of hospitalized patients with AMI after PCI, and it can narrow the prognostic gap still existing between DM and non-DM patients (65).

#### 3.1.2 Reduce the harmful effects of advanced glycation end-products

Advanced glycation end-products (AGEs), called glycation or Maillard reactions, are chemicals that result from non-enzymatic interactions between proteins and sugar residues (66). AGEs are recognized by a variety of cellular receptors in the body and trigger many signaling pathways associated with inflammation and oxidative stress (66). AGEs continue to accumulate in the body as we age, and DM accelerates the development of this process (67). The accumulation of AGEs resulted in increased expression of receptors for AGEs (RAGE), inflammatory cell count, intercellular adhesion molecule (ICAM)-1, and vascular cell adhesion molecule (VCAM)-1 (68, 69). The engagement of RAGE with AGEs elicits oxidative stress generation and subsequently induces the proliferation, adhesion, and migration of SMC, thus evoking vascular injury in diabetes (68). These findings may verify that the accumulation of AGEs may contribute to the increased rate of restenosis in people with DM (50, 70).

An animal and cell experiment showed that empagliflozin reduced AGEs in serum by inhibiting the AGEs-RAGE pathway, inhibiting the expression of the RAGE, and at the same time reducing levels of cholesterol as well as proteins associated with cholesterol metabolism (71). Empagliflozin also plays an anti-inflammatory and antifibrotic role in diabetic nephropathy by inhibiting the AGEs-receptor axis (72). Dapagliflozin protects podocytes from advanced AGEs-induced inflammatory response and apoptosis via the AMPK/mTOR (mammalian target of rapamycin)-mediated autophagy pathway (73). The above studies suggest that SGLT2 inhibitors may play an anti-atherosclerotic role by inhibiting AGEs, but it needs to be further validated in vascular models.

#### 3.1.3 Attenuate insulin resistance

Insulin resistance (IR) is a significant risk factor for CHD and is associated with inflammatory responses (74), metabolic disorders (75), elevated fibrinogen and decreased plasminogen activator (74), and endothelial dysfunction (76), leading to adverse cardiovascular events (61, 77). IR is also associated with the incidence of restenosis (75, 78–80). Adipose tissue plays a key role in the pathogenesis of IR and DM through the secretion of adipokines and inflammatory cytokines, as well as the accumulation of visceral fat (81, 82). Dysfunction and reduced metabolism of fat cells are strongly associated with IR and DM, although their role in IR is complex and poorly understood.

Drugs can lead to adaptive responses in glucose homeostasis and hormone release, and the genetic or pharmacological inhibition of SGLT2 has positive effects on hyperinsulinemia and hyperglycemia (83, 84). At present, SGLT2 inhibitors has been shown to significantly attenuate insulin resistance. The mechanism by which SGLT2 inhibitor attenuates IR can be summarized as follows: (1) by regulating the body's energy balance and metabolism, (2) by promoting the transformation of white adipose tissue into brown adipose tissue, and (3) by inhibiting the inflammation induced by polarizing M2 macrophages (85–90).

### 3.2 SGLT2 inhibitor can inhibit neointima formation

As a result of stent implantation, the physiological barrier function of EC is impaired due to changes in cell connectivity, and its ability to regulate vascular tone, control inflammation, maintain lipid and tissue fluid homeostasis, and prevent thrombosis is reduced (91, 92). At the same time, SMC changed from contractive phenotype to synthetic phenotype (93). The proliferation and migration of SMC were enhanced, and the secretion of cytokines and extracellular matrix was increased. Synthetic SMC can also migrate into the lipid plaque adhering to the intima to phagocytose lipids, form foam cells, synthesize and secrete fibrin, and eventually cause ISR (94).

SGLT2 inhibitors can effectively regulate the proliferation, migration, differentiation, survival, and senescence of EC (95). The DEFENCE study was the first to assess the role of dapagliflozin on vascular endothelial function in T2DM patients (96). Flow-mediated dilation (FMD) is recognized as an alternative marker of early endothelial dysfunction. dapagliflozin combined with therapy on metformin improved endothelial function in patients with inadequate glycemic control assessed by FMD. Sawada et al. found that empagliflozin can also increase FMD% and play an endothelial protective role by improving hypertriglyceridemia and IR (97). In addition, SGLT2 inhibitor also inhibits the contraction of VSMC and blocks the proliferation and migration of these cells. Hashikata et al. found that standard therapy add-on empagliflozin reduced neointimal progression compared to intensive standard therapy after DES implantation (98). Therefore, SGLT2 inhibitors might be promising for the prevention of ISR following PCI, especially in DM patients at high risk for restenosis.

#### 3.2.1 Inhibit the expression of SGLT2 in endothelial cells and smooth muscle cells

It was previously agreed that SGLT2 is mainly expressed in human kidney and small intestine cells (29), but could not be detected in human myocardium (30). Recently, SGLT2 was found to be expressed in human endothelial cells (EC) and smooth muscle cells (SMC) using immunocytochemistry and western blotting techniques (31, 32). Interestingly, D'Onofrio N. et al. found that plaques from patients with diabetes had higher SGLT2 expression, inflammation, and oxidative stress, compared to plaques from patients without diabetes (63). This seems to be consistent with a higher risk of coronary atherosclerotic heart disease and restenosis in people with diabetes. Dutzmann et al. demonstrated that empagliflozin damages SMC proliferation and accelerates endothelial regeneration *in vitro*. In addition, empagliflozin prevents neointimal lesion formation and enhances reendothelialization after vascular injury *in vivo* (31). The mechanism may depend on vascular SGLT2 and involve the cardiovascular active peptide apelin.

Recently, dapagliflozin has also been shown to have a unique potential for cardioprotective effects by modulating apelin levels in patients and may serve as an alternative target for HF management in T2DM patients (99). Apelin is involved in the negative regulation of SMC proliferation and the improvement of EC function (100, 101), but the specific mechanism is not clear. However, the specific role of EC and SMC expression of SGLT2 is still unclear, and more studies may be needed to confirm and explore its mechanism of action in the future.

#### 3.2.2 Enhance eNOS activation and increase the bioavailability of NO in endoderm

The release of NO by endothelial nitric oxide synthase (eNOS) plays an important role in maintaining vascular health (102). NO has been demonstrated to decrease migration and proliferation of VSMCs to attenuate the binding of inflammatory cells to the vascular wall, inhibit thrombosis by reducing platelet adhesion and aggregation, and resist angiosclerosis (103, 104). The regenerated vascular endothelial cells not only had poor cell junction formation but also significantly decreased the expression of antithrombotic molecules and NO (80). Disruption of NO synthesis leads to endothelial dysfunction and loss of homeostasis, resulting in endothelial cell apoptosis, enhanced endothelial permeability, impaired endothelium-dependent vasodilation, endothelial cell activation, inflammation, thrombosis, and neointima formation, which contribute to the development of vascular disease (105). Restenosis is closely related to the decrease of NO production by the damaged EC and the decrease in the amount of enzyme protein produced by the EC (106). Furthermore, Wu et al. showed that NO-dependent endothelial vasodilation was impaired in patients with ISR after coronary stent placement, compared with patients without restenosis (107).

SGLT2 inhibitor can enhance eNOS activation, increase the bioavailability of endodermal NO, and restore endothelium-dependent vasodilation (108, 109), these might have contributed to the beneficial vascular effects. A prospective cohort study demonstrated that empagliflozin increases blood viscosity and carotid shear stress (SS) as well as decreases carotid wall thickness (110). Within a certain range, higher levels of wall SS have a protective effect on atherosclerosis, which is conducive to the release of NO and the regulation of endothelial function and hemodynamic performance. In empagliflozin-treated mice, the activity of NO synthase in vascular endothelium increased, the content of phosphorylated cofilin and filamentous actin decreased, and the pathway involved in ROS production was down-regulated (109). Salim et al. found that ipragliflozin administration ameliorated impaired phosphorylation of eNOS, and attenuated endothelial dysfunction in diabetic mice (111). Therefore, substantial evidence supports the role of SGLT2 inhibitor in maintaining NO bioavailability and endothelial function by influencing the activity or expression of many molecules involved in eNOS, oxidative stress, inflammation, and blood pressure. The endothelial protection function of SGLT2 inhibitor plays an important role in delaying the formation of ISR.

### 3.3 Inhibit inflammation and oxidative stress

Inflammation is involved in the occurrence and development of atherosclerosis and affects its prognosis. There is increasing evidence that low-grade chronic inflammation is a key link in the pathogenesis of ISR (112). Endothelial injury after PCI causes inflammatory activation, thrombosis, and proliferation of vascular smooth muscle cells, and eventually leads to ISR formation (66). Inflammatory markers such as interleukin (IL)-6, IL-8, tumor necrosis factor (TNF) -α, monocyte chemotactic protein (MCP) -1, and matrix metalloproteinase (MMP) have been shown to play important roles in the pathogenesis of coronary atherosclerotic heart disease and ISR (113, 114). It can be used as a predictor of prevention and treatment of intra-stent restenosis. By monitoring changes in inflammation-related indicators, early identification of patients with ISR can be achieved, reducing the risk of sudden death in patients with coronary atherosclerotic heart disease.

The increased expression of SGLT2 was associated with higher oxidative stress, nuclear factor-κ B (NF- κ B), and proinflammatory cytokines (63). SGLT2 inhibitor treatment significantly reduces the expression of inflammatory markers TNF-α, IL-1β, and IL-6, thereby delaying the occurrence and progression of atherosclerosis (115). An international multicenter observational study found that in patients with diabetes who underwent PCI, the use of SGLT2 inhibitor was an important predictor of reduced inflammatory response (43). And recent studies have found that SGLT2 inhibitor reduces infarct size and the occurrence of ischemia-reperfusion-induced arrhythmias, and plays a cardioprotective role, which may be achieved by inhibiting inflammatory factors and oxidative stress (116). Therefore, SGLT2 inhibitor could significantly reduce the inflammatory index in patients after PCI, improving the long-term prognosis.

#### 3.3.1 Reduce ROS generation

Inflammation and oxidative stress are both important and interdependent factors in almost all major cardiovascular diseases (117). Inflammation can induce oxidative stress directly or indirectly through a variety of cell signaling pathways, including mediators that activate ROS sources. Several molecular mechanisms have been implicated in hyperglycemic-induced endothelial damage, such as activation of PKC isoforms, increased hexosamine pathway flux, increased AGEs formation, increased polyol pathway flux, and activation of the proinflammatory NF-k B, which are associated with overproduction of ROS (19, 52, 53, 58, 118). In addition, excess glucose can be metabolized through the pentose phosphate pathway and produce nicotinamide adenine dinucleotide phosphate hydrogen (NADPH) (119). NADPH is a substrate for cytoplasmic NADPH oxidase, an enzyme complex that can produce ROS (117). Eventually, ROS can lead to endothelial dysfunction, decreased vasodilation, and atherosclerotic plaque formation, which will lead to coronary heart disease and restenosis.

Studies have shown that SGLT2 inhibitor can lead to downregulation of key proteins and pathways involved in ROS biosynthesis (109, 120). Li et al. found that SGLT2 inhibitor has an antioxidative effect and alleviates mechanical forces induced endothelial permeability in human coronary artery endothelial cells, which may be mediated by ROS clearance (121). In addition, they found that the antioxidative effect of SGLT2 inhibitors might be mediated in part by inhibition of NADPH oxidase and Na + /H + exchanger (NHE). Elevated intracellular Na + hinders mitochondrial Ca2 + processing and deteriorates energy supply and demand matching and the mitochondrial antioxidative defense systems (122). By decreasing the intracellular Na + concentrations through inhibition of the mitochondrial NHE, the production of ROS can be inhibited, thus having a favorable effect on cardiac metabolism (123, 124). Of note, NHE is also expressed in VSMC, and inhibition of NHE blunts lysophosphatidic acid-induced VSMC proliferation (125). Dapagliflozin and empagliflozin attenuate the upregulation of the cardiac NHE in mouse cardio fibroblasts stimulated with lipopolysaccharides (126, 127). The effect of dapagliflozin was blocked with an AMPK inhibitor, and it suggested that dapagliflozin may inhibit NHE by inhibiting the expression of AMPK, exerting the cardiovascular protective effect of antioxidative stress. However, in the previous studies, AMPK activation was not only observed in empagliflozin and dapagliflozin but in canagliflozin-treated cells (120). It seems paradoxical, and the involvement of AMPK phosphorylation may require further discussion.

#### 3.3.2 Inhibit Nf-**κ** B pathway

The nuclear factor-κ B family of transcription factors, especially p65 and p50, participate in the pathogenesis of vascular proliferative diseases (128). Expression of NF-κ B has also been detected in the nucleus of human atherosclerotic lesions. We have previously demonstrated in restenosis animal models that inflammation induced by the NF-κ B pathway is closely relevant to the occurrence and development of coronary atherosclerosis and vascular restenosis after interventional therapy (129). Our results showed that the inhibition of NF-κ B pathway can downregulate the expression of downstream factors IL-1, IL-6, TNF-α, VCAM-1, and MCP-1, which can significantly improve the lipid profile and inhibit restenosis.

SGLT2 inhibitor may reduce the gene expression level of inflammatory factors and protein expression level of NF-κ B, thus alleviating atherosclerosis and ISR progression. Mice treated with dapagliflozin were found to have reduced atherosclerotic plaques and reduced serum levels of inflammatory markers such as IL-6, IL-8, TNF-α, and MCP-1 (130). Gaspari et al. also found that dapagliflozin-mediated attenuation of TNFα-induced NF-κ B mRNA expression implicates dapagliflozin in transcriptional regulation (131). Similarly, empagliflozin can reduce the gene expression level of inflammatory factors and protein expression level of NF-κ B, improve the protein expression level of AMPK affected by ox-LDL in cells, and delay the formation of atherosclerosis (132).

#### 3.3.3 Inhibit NLRP3 pathway

Previous studies revealed elevated expression of the nucleotide binding domain-like receptor protein-3 (NLRP3) inflammasome in human atherosclerotic arteries and restenosis artery wall (133, 134). A study showed that NLRP3 binding to ligands promotes inflammasome formation, activates caspase-1, and eventually promotes the maturation and secretion of IL-1β, and IL-18 (135). Those cytokines are induced following vascular injury and play a role in intimal hyperplasia. Notably, compared with those of the non-DM mice, DM mice had significantly increased serum levels of NLRP3, IL-1β and IL-18 (136). Recent studies have shown that targeted inhibition of NLRP3 can dampens inflammatory leucocyte production and uptake in atherosclerosis, significantly enhancing reendothelialization and prevent neointimal formation (137, 138). It suggests that NLRP3 inflammasome may be a promising therapeutic target for restenosis.

In vitro and *in vivo* experiments have demonstrated that SGLT2 inhibitor might inhibit the NLRP3 inflammasome, thereby reducing the secretion of inflammatory markers. In addition, under normal glucose conditions, SGLT2 inhibitor inhibits SMC migration and proliferation by targeting IL-17A mediated IL-1β and IL-18 expression, NLRP3 expression, and inflammatory responses (32, 139). Leng et al. elucidate that dapagliflozin treatment was associated with the inhibition of the secretion of IL-1β by macrophage via the ROS-NLRP3-caspase-1 pathway in atherosclerosis for the first time (136). In addition, empagliflozin can degrade the inflammatory component NLRP3 through selective autophagy and reduce the maturation and secretion of inflammatory factors (140). SGLT2 inhibitor has been demonstrated to cause a considerable increase in serum β-hydroxybutyrate with a parallel decline in fasting serum insulin levels due to a significant improvement in insulin sensitivity (141). These effects were associated with decreased IL-1β production and inhibition of NLRP3 inflammasome activity, which might help explain its cardioprotective effects.

### 3.4 SGLT2 inhibitor can regulate lipid metabolism

Drug therapy strategies based on statins and supplemented by a variety of lipid-lowering drugs, including PCSK9 inhibitors, have been widely used in secondary prevention of atherosclerotic cardiovascular disease (142). Although this optimized drug therapy reduces the risk of cardiovascular disease, there is still a residual risk that cannot be controlled. The effect of dapagliflozin combined with statins on reducing triglyceride-glucose (TyG), a related indicator of glycolipid metabolism, was significantly better than that of statins alone (42). This suggests that the target of SGLT2 inhibitor may not be limited to glucose metabolism, but also has a regulatory effect on lipid metabolism.

#### 3.4.1 Regulate the secretion of adipokines

It is known that the adipose tissue can produce and secrete a wide variety of bioactive molecules known collectively as adipocytokines, such as adiponectin (APN), leptin, IL-6, IL-1β, and MCP-1 (143, 144). Those adipocytokines are not only involved in fatty acid metabolism and glucose homeostasis but also play a role in the control of inflammatory responses (145). Adipose tissue-specific adipocytokines leptin and APN interact with AMPK to regulate fatty acid and energy metabolism (146).

In our previous study (147), we established a rabbit model of iliac artery restenosis by percutaneous intracavity balloon angioplasty. Our previous research has shown that the expressions of TNF-α, IL-6, and MCP-1 were considerably increased (*P* < 0.01), while the expression of APN in the restenosis group was considerably decreased (*P* < 0.01). We conclude that balloon angioplasty and high-fat diet can inhibit APN expression and increasing APN expression may prevent the formation of restenosis after PCI (148). In atherosclerotic ApoE(-/-) mice, changes in adipokines were consistent with our data. After treatment with empagliflozin, the circulating concentrations of TNF-α, IL-6, and MCP-1 were decreased, and APN concentrations were increased, which played an anti-atherosclerotic role (149). Interestingly, changes in these adipocytokine levels were closely related to changes in blood lipids, IR, and body weight. Compared with sitagliptin, another glucose-lowering agent, dapagliflozin can significantly increase serum APN levels in diabetic patients (150). Nishitani et al. performed gene expression microarray and metabolomic analyses of mouse adipose tissue, and the result showed that dapagliflozin improved serum glucose levels and induced 3-hydroxybutyric acid in diabetic mice (87). 3-hydroxybutyric acid has been proven to enhance adiponectin expression in adipocytes, and thus exert anti-inflammatory and anti-atherosclerosis effects (87). In addition, leptin also plays an important role in the regulation of vascular function. It has been reported that leptin can regulate the synthesis of NO in ECs and play a vascular protective role (151). Elevated leptin levels were present in patients with restenosis after stent placement, while consistent results were confirmed in patients without restenosis and in the control group (80). Recently, a meta-analysis concluded that SGLT2 inhibitors treatment was associated with decreased serum leptin levels and increased serum APN levels, which may contribute to the positive effects of SGLT2 inhibitors on metabolic homeostasis (152).

#### 3.4.2 Regulate plasma lipid profiles

Clinical data and animal studies have shown that lipid levels are closely related to the occurrence of CHD and restenosis. It has been demonstrated that an increase in high-density lipoprotein cholesterol levels may be a predictor of anti-atherosclerotic effect (98). We have also verified in previous studies that restenosis is often accompanied by increased plasma levels of total cholesterol, low density lipoprotein cholesterol, and very low-density lipoprotein cholesterol (129, 147). Recent studies have found that improvement in hypertriglyceridemia was the strongest independent predictor of improvement in endothelial function in CAD patients with diabetes (97). There was an independent positive correlation between the TyG index and the risk of ISR in acute coronary syndrome patients after DES implantation (153).

SGLT2 inhibitor has been shown to exert a cardiovascular protective effect by affecting plasma lipid levels (21). In comparison with sitagliptin, dapagliflozin decreases low-density lipoprotein cholesterol and increases high-density lipoprotein cholesterol in patients with type 2 diabetes (150). The EMPAREG OUTCOME study also showed a sustained increase in high-density lipoprotein cholesterol levels less than 6 months after taking empagliflozin as well. In addition, empagliflozin has also been shown to play a protective role in vascular endothelium by improving hypertriglyceridemia in patients with coronary heart disease and diabetes (97).

### 3.5 Inhibit the sympathetic nervous system

There is strong evidence implicating sympathetic nervous system activation in the pathogenesis of cardio-metabolic illnesses including obesity, metabolic syndrome (MetS), diabetes, and hypertension (HTN) (154). The rapid increase in sympathetic nerve activity leads to increased levels of lipolysis and fatty acids in plasma, increased gluconeogenesis in the liver, inhibition of insulin release, and a shift in fuel metabolism in the muscles towards fatty acid oxidation and other directions (155, 156). However, if the sympathetic activity is chronically elevated due to a poor lifestyle, the physiological responses may move in an unfavorable direction, including elevated fasting blood glucose levels and IR (157), as well as elevated blood pressure and hypertension (158).

A recent study demonstrated that canagliflozin increased energy expenditure and fat utilization by increasing sympathetic activation of adipose tissue (159). Herat et al. also found that dapagliflozin-treated MetS mice fed a high-fat diet showed reduced blood pressure, weight loss, reduced hyperglycemia, and increased glucose tolerance (22). Interestingly, dapagliflozin treated high-fat diet mice, reducing renal tyrosine hydroxylase and norepinephrine levels in MetS mice. This suggests that dapagliflozin inhibition of SGLT2 leads to metabolic benefits in MetS mouse models via sympathetic inhibition. Matthews et al. found that SGLT2 expression increased in human renal proximal tubule cells after treated with norepinephrine for 48 h (157). The data support that sympathetic nervous system activation may up-regulate SGLT2 expression in human renal proximal tubule cells. The EMBODY trial (21) elucidates the mechanism by which empagliflozin reduces cardiovascular-related death, including sudden cardiac death in patients with AMI and T2DM. The trial identified that SGLT2 inhibitor may show significant improvements in cardiac sympathetic and parasympathetic nerve activity in heart rate variability and heart rate turbulence, and this mechanism also plays a beneficial role in the prognosis of patients after PCI.

## 4 The safety of SGLT2 inhibitors

Safety is the major concern after the emergence of new drugs, and attention should be paid to any problems that may arise. Known allergic reactions, pregnancy/lactation period, eGFR <20 ml/ min / 1.73 m^2^, symptoms of hypotension or systolic blood pressure <95 mmHg are contraindications for SGLT2 inhibitors (160). Adverse events might occur with SGLT2 inhibitors including an increased risk of orthostatic hypotension, urinary tract infection, diabetic ketoacidosis, renal impairment, and possibly limited amputations (161–163). Current guidelines and expert consensus generally agree that SGLT2 inhibitors should be avoided whenever possible during acute progression to avoid adverse events (164). However, in some clinical trials on acute and chronic HF, these adverse events were uncommon in the SGLT2 inhibitors treatment group, and the incidence of adverse events was not significantly increased compared to the control group (165, 166). Overall, SGLT2 inhibitors has many positive effects, and the benefits of using SGLT2 inhibitors far outweigh the risks. In addition, these adverse events are preventable and ameliorable, and these adverse reactions should not be used as contraindications for the use of the drug.

### 4.1 Orthostatic hypotension

Treatment with SGLT2 inhibitors reduces volume and sodium load through its diuretic and natriuretic properties. The resulting reduction in circulatory load, especially in ventricular filling pressure and cardiac load, may be an important mechanism by which the use of SGLT2 inhibitors can reduce mortality in patients. There was evidence that patients treated with empagliflozin and dapagliflozin had similar rates of hypotension and other adverse events related to volume reduction compared with placebo (165). However, in the CANVAS program, patients using canagliflozin showed a significant difference in volume reduction compared to placebo (26.0 vs. 18.5 event rates per 1,000 patient years, *p* = 0.009) (40). When symptomatic hypotension or a systolic blood pressure <95 mmHg, patients are prohibited from using SGLT2 inhibitors (160). Therefore, the volume status of patients should be assessed and treated before starting SGLT2 inhibitors. Caution should be exercised when initiating SGLT2 inhibitors in patients with decreased kidney function, older adults, and low baseline systolic blood pressure. During medication, blood pressure should be monitored and the dosage of both diuretics and drugs that affect blood pressure should be adjusted.

### 4.2 Urinary tract infection

Because SGLT2 inhibitors can cause significant glycosuria, especially in patients with T2DM, there is an increased risk of genital infection. Although meta-analyses have found that SGLT2 inhibitors do not increase the occurrence of urinary tract infections, these results may contain additional sources of uncertainty (167). To avoid urinary tract infection risk, prior to the use of SGLT2 inhibitors, possible previous urogenital infections and existing risk factors should be documented. In addition, SGLT2 inhibitors are not recommended for patients with recurrent genitourinary infections within 6 months.

### 4.3 Euglycemic diabetic ketoacidosis (EDKA)

SGLT2 inhibitors produces ketone bodies in the presence of normal blood sugar levels (168). Recent studies have suggested that the increase in ketone bodies produced by SGLT2 inhibitors creates a “super fuel” that can improve the efficiency of the heart and kidneys, thereby improving cardiac contractility and kidney function (169). Although increased ketone body formation can provide potential benefits for heart and kidney function, increased ketone body production will continue to put patients at increased risk for ketoacidosis (170). Patients with abdominal pain, nausea, vomiting, weakness, and dyspnea should be considered for EDKA during SGLT2 inhibitor use. Once EDKA is diagnosed, SGLT2 inhibitors should be discontinued. In addition, the ESC guide recommends that type 1 diabetes mellitus is not an absolute contraindication, but an individual risk of ketoacidosis should be considered when starting SGLT2 inhibitors therapy (160).

### 4.4 Amputation

The CANVAS study showed that canagliflozin increased the risk of lower limb amputation, while other studies have not confirmed whether SGLT2 inhibitor treatment is associated with lower limb amputation (171). Canagliflozin can damage the proliferation and migration of endothelial cells and smooth muscle cells, which may affect the re-endothelialization of arteries after PCI and play a positive role in the prevention of ISR (172, 173). On the other hand, it may exacerbate peripheral artery disease in diabetic patients by restricting angiogenesis. SGLT2 inhibitors should be used with caution in patients with risk factors for amputation, especially those with a previous history of amputation or foot ulcers, neuropathy, or peripheral vascular disease.

## 5 Conclusion and prospect

In summary, SGLT2 inhibitors can improve the prognosis of patients after PCI by improving glucose and lipid metabolism, inhibiting inflammatory response and oxidative stress, and improving vascular endothelial function and vascular remodeling. In addition, the early initiation of SGLT2 inhibitors can reduce the myocardial infarction size and the occurrence of reperfusion injury during AMI and can improve further outcome of impaired left ventricular function after AMI. SGLT2 inhibitors may be a new hope to improve the prognosis of patients with coronary heart disease, especially those with myocardial infarction, and prevent the recurrence of cardiovascular events. Recent studies suggest that the cardiovascular protective effect may remain significant in non-DM patients, independent of its hypoglycemic effect. In the future, more randomized controlled trials are needed to verify the beneficial effect of SGLT2 inhibitors in patients with coronary heart disease to to address the residual risks of current treatment options.
